# Uninsured Patients Under Dialysis in Greece: A Case-Control Study

**DOI:** 10.7759/cureus.97723

**Published:** 2025-11-25

**Authors:** Ioanna Revela, Vasiliki Gkika, Eleni Gkogka, Despina Smirloglou, Christos Pallas Nikolov, Sofia Palla, Eleni Chelioti, Rigas G Kalaitzidis

**Affiliations:** 1 Dialysis Center, Frontis, Piraeus, GRC; 2 Center for Nephrology "G. Papadakis", General Hospital of Nikaia "Agios Panteleimon", Piraeus, GRC; 3 Department of Internal Medicine, Thriasio General Hospital of Elefsina, Athens, GRC; 4 Department of Nephrology, Thriasio General Hospital of Elefsina, Athens, GRC; 5 Department of Nephrology, Tzaneio General Hospital of Piraeus, Piraeus, GRC

**Keywords:** dialysis, esrd, health policies, immigrants, uninsured

## Abstract

Introduction: Treating uninsured patients with end-stage renal disease (ESRD) undergoing dialysis is a challenge for every country. Greece has adopted a three-week scheduled outpatient over emergency dialysis for uninsured patients with ESRD from the pandemic until now. In this observational study, we describe the experience of treating uninsured ESRD patients in a private dialysis unit in Greece.

Methods: A total of 50 patients who underwent dialysis at Frontis Dialysis Center, Piraeus, Greece, were analyzed. Clinical and laboratory data were prospectively collected from 25 uninsured consecutive patients and compared with 25 age- and sex-matched insured dialysis patients who served as controls.

Results: The uninsured population was treated with three-week scheduled dialysis session. The mean age of the study population was 44.4 years (SD=13.5), and 68% were male. At presentation, 60% of uninsured patients were treated with hemodialysis via central venous catheter, and hemodiafiltration dialysis was applied only in 20% of them (p-value <0.001). Initially, the median hemoglobin (9.8 g/dL (min-max: 8-12.1) versus 11 g/dL (min-max: 9-14), p-value=0.04) and albumin (3.6 g/dL (min-max: 2-4) versus 4 g/dL (min-max: 3.3-4.6), p-value=0.008) levels were significantly lower, while the median ferritin (518 ng/mL (min-max: 104-2,149) versus 208 ng/mL (min-max: 16-1,333), p-value <0.001) and parathormone (iPTH) levels (799 pg/dL (min-max: 168-2,811) versus 410 pg/dL (min-max: 13-923), p-value <0.001) were significantly higher for the uninsured patients. After three months, uninsured patients improved their anemia and nutritional status, but ferritin and iPTH levels were still significantly higher. At the end of follow-up, there were no differences between the two groups. Rates of hospitalization (40% versus 32%, p-value=0.5) and infections (24% versus 12%, p-value=0.2) did not differ between groups. During the study, there was only one cardiovascular event in the control group, and none in the uninsured group of patients. None of the patients died during the study.

Conclusions: Uninsured patients who received three-week scheduled dialysis sessions improved all their laboratory values and had zero mortality. Healthcare policy has to take into account that scheduled dialysis sessions result in favorable outcome for uninsured patients.

## Introduction

Uninsured patients with end-stage renal disease (ESRD) requiring dialysis constitute a worldwide issue for health systems [1,2]. Undocumented immigrants are a great proportion of the uninsured population. Relative to the population, in 2024, Greece was in the top three European countries with the highest number of first-time asylum applications from immigrants [3]. During 2024, 53,270 irregular arrivals were recorded in the European Union. The Eastern Mediterranean route refers to irregular arrivals in Greece [4].

In many states of the United States of America (USA), uninsured individuals with ESRD receive only emergency dialysis [5]. However, scheduled dialysis was associated with better survival, decreased acute care utilization, and decreased costs over one year [6-9].

In Greece, during the COVID-19 pandemic, uninsured patients with ESRD under dialysis were allocated to private dialysis centres for scheduled hemodialysis(HD) [10]. Therefore, the uninsured population with the need for renal replacement therapy included immigrants/refugees and Greek-born unemployed patients.

Several conflicts about financial and medical issues arise while treating uninsured ESRD dialysis-dependent patients in different countries [1,2,5]. Our hypothesis was that uninsured dialysis patients are more likely to experience poorer clinical outcomes due to socioeconomic and system-level barriers rather than underlying medical factors. The primary aim of the study was to determine how insurance status influences clinical outcomes and quality of care among individuals undergoing dialysis in Greece. Therefore, we examined differences in laboratory parameters at baseline and throughout follow-up, assessed differences in dialysis modality and vascular access type, and finally examined morbidity and mortality outcomes over the study period between the uninsured patients and a cohort of age- and sex-matched insured dialysis patients who served as controls.

## Materials and methods

Study population

We prospectively collected data from all consecutive uninsured patients who initiated or continued maintenance hemodialysis at our dialysis unit between January 1, 2023, and December 31, 2024. A total of 25 uninsured patients met the inclusion criteria. Eligible participants were adults aged >18 years with established ESRD receiving thrice-weekly outpatient hemodialysis, with each session lasting approximately four hours. Patients were excluded if they required dialysis due to acute kidney injury or if their medical records did not contain sufficient clinical or laboratory data for analysis.

For each uninsured patient, one insured patient matched on age (±3 years) and sex was selected from the same dialysis unit during the same time period, resulting in 25 insured controls. Matching was performed prior to data extraction to minimize selection bias.

Follow-up assessments occurred at baseline, three months after study entry, and at the end of the study period. Demographic and clinical data were recorded at baseline by the treating nephrologist and included age, sex, country of origin, history of hypertension, diabetes mellitus, cardiovascular disease, smoking status, dialysis vintage, and vascular access type at dialysis initiation. Insurance status was verified through electronic administrative records.

Laboratory parameters collected at baseline and longitudinally included complete blood count, serum creatinine, urea, electrolytes (sodium and potassium), albumin, calcium, phosphorus, ferritin, and intact parathyroid hormone (iPTH). Hospitalizations, dialysis-related infections, and mortality events were recorded throughout the follow-up period using unit medical records and hospital discharge summaries. The same data collection protocol and follow-up schedule were applied identically to both groups.

A minimum required sample size of 16 cases and 16 matched controls was calculated (α = 0.05, power = 0.80) using the Cleveland Clinic Risk Calculator Library, confirming adequacy of the final sample for detecting clinically meaningful differences. The study was conducted at Frontis Dialysis Center, Piraeus, Greece, and approved by the ethics committee of our institution.

Statistical analysis

Results were expressed as mean (standard deviation) for normally distributed continuous variables, as median (min-max) for skewed variables, and as frequency (%) for categorical variables. Deviations from normal values were tested with the use of the Kolmogorov-Smirnov (K-S) test. Group differences in continuous variables were tested with the use of Student's t-test and Mann-Whitney, and Wilcoxon signed rank in the case of deviations from normal values. Dependence tests between discrete variables were performed with the use of the chi-square test and Fisher's exact test, where appropriate. A p-value<0.05 (two-tailed) was considered statistically significant. All analyses were performed with the statistical software package STATA version 13.0 (StataCorp LLC, College Station, TX).

## Results

The mean age of the study population was 44.4 years (SD=13.5), while 68% were male (n=34). The uninsured population included mostly immigrants (n=21, 84%), while Greek-born unemployed patients were a minority (n=4, 16%). The top three nationalities of irregular arrivals were Somalian, Syrian, and Palestinian. The baseline patient’s characteristics are presented in Table 1. The median period of stay for immigrants was 3.2 (min-max: 3-11) months. A history of hypertension and smoking was more frequent in insured patients (Table 1).

**Table 1:** Baseline characteristics of patients (pts) ^#^ AVF or AVG =arteriovenous fistula or arteriovenous graft; ^$ ^Wilcoxon signed rank for continuous variables and the chi-square test or Fisher's exact test for discrete variables.

Demographics	Uninsured pts N=25	Insured pts N=25	p-value^$^
Age, mean (SD)	42 (17)	46 (8.5)	0.15
Sex, male (n, %)	18 (72)	16 (64)	0.54
Race Greek (n, %)	4 (16)	25 (100)	< 0.001
Vascular access during enrolment			
Central Venous Catheter (n, %)	15 (60)	8 (32)	0.04
AVF or AVG^#^ (n, %)	10 (40)	17 (68)	0.04
History			
Hypertension (n, %)	15 (60)	23 (92)	0.008
Diabetes (n, %)			
Type 1 (n, %)	2 (12)	3 (8)	0.63
Type 2 (n, %)	1 (4)	5 (20)	0.08
Smoke (n, %)	3 (12)	12 (48)	0.005
Cardiovascular disease (n, %)	0 (0)	2 (8)	0.14

Hemodialysis access and modality

At presentation, the majority (60%) of uninsured patients were treated with hemodialysis via a central venous catheter (CVC). In comparison, in the matched control group of insured patients, 68% were treated via arteriovenous fistulas and 32% via a CVC (Table 1). Surgery for the creation of arteriovenous access was offered in both groups in Greece and was performed in eight out of 10 uninsured patients and nine out of 10 insured patients with a CVC (p-value=0.76).

Online hemodiafiltration dialysis (HDF) was applied in the majority of the insured group (n=21, 84%), while only 20% uninsured individuals underwent online HDF dialysis (p-value <0.001).

Laboratory values

At the presentation, the median hemoglobin (p-value=0.04) and albumin levels (p-value=0.008) were significantly lower for the uninsured patients compared to the matched control group (p-value=0.04, Table 2), while the median ferritin levels and iPTH levels at initiation were significantly higher (p-value <0.001) (Table 2). In the uninsured group, the median creatinine levels (p=0.005) and potassium levels (p=0.01) were higher, while sodium levels were lower (p=0.01). There were no differences in urea, calcium, phosphorus, and albumin levels between the two groups (Table 2).

**Table 2:** Laboratory levels at presentation Data are expressed as median (min-max). ^# ^Wilcoxon signed rank

Parameter	Uninsured pts N=25	Insured pts N=25	p-value^#^
Hematocrit, (%)	29 (23-38.5)	33 (25-43)	0.04
Hemoglobin, g/dL	9.8 (8-12.1)	11 (9-14)	0.04
Urea, mg/dL	139 (66-221)	152 (75-202)	0.28
Creatinine, mg/dL	11 (4.6-17)	7 (4-13)	0.005
Potassium, mmol/L	5 (3.6-6.9)	4.3 (3.3-6.2)	0.01
Sodium, mmol/L	136 (124-143)	139 (134-143)	0.01
Calcium, mg/dL	8.4 (6.6-10)	8.7 (7.4-10)	0.12
Phosphorus, mg/dL	4.5 (1.9-8.7)	5.4 (3.5-8.4)	0.09
Parathormone, pg/dL	799 (168-2811)	410 (13-923)	<0.001
Ferritin, ng/mL	518 (104-2149)	208 (16-1333)	<0.001
Albumin, g/dL	3.6 (2-4)	4 (3.3-4.6)	0.008

**Figure 1:**
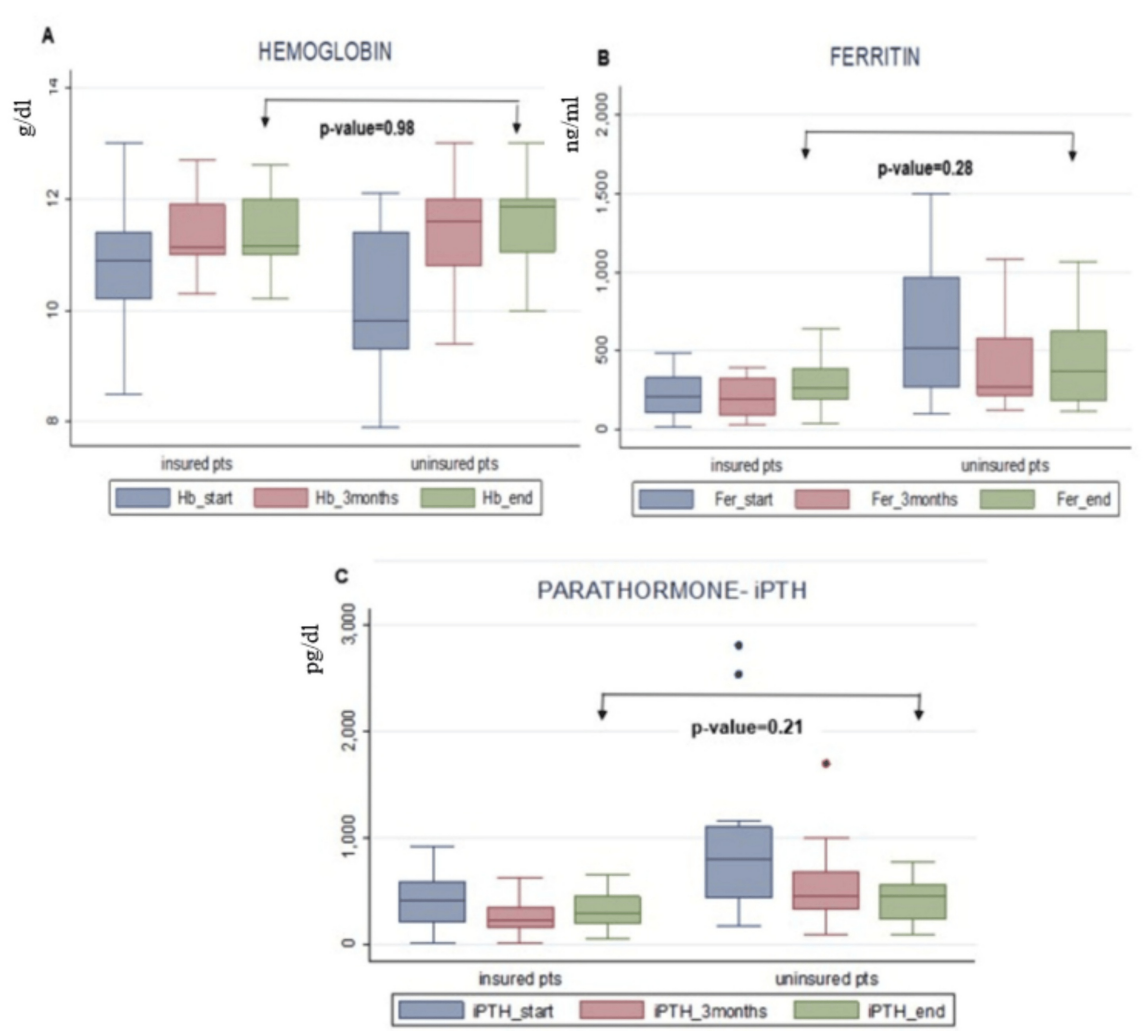
Box plots of hemoglobin, intact parathyroid hormone (iPTH), and ferritin levels between uninsured dialysis patients and matched control insured patients A. Hemoglobin, B. ferritin, and C. iPTH levels

However, during the three-month follow-up, anemia was improved in the uninsured group, as well as their iPTH and ferritin levels, but their values remained significantly higher compared to the control group (Table 3). There were no differences in hemoglobin, potassium, sodium, calcium, and phosphorus levels between the two groups (Table 3). At the end of the follow-up period, there were no differences between the two groups, as shown in Figure 1.

**Table 3:** Laboratory levels after the three-month follow-up Data are expressed as median (min-max); ^# ^Wilcoxon signed rank

Parameter	Uninsured pts N=25	Insured pts N=25	p-value^#^
Hematocrit, (%)	36 (29-42)	34 (33-38)	0.18
Hemoglobin, g/dL	11.6 (9.4-14)	11(10-14)	0.38
Urea, mg/dL	131 (75-198)	138 (105-190)	0.32
Creatinine, mg/dL	9.7 (4-14)	7.7 (4.2-14)	0.007
Potassium, mmol/L	4.7 (3.9-5.9)	4.5 (3.6-6)	0.30
Sodium, mmol/L	138 (128-141)	137(133-143)	0.82
Calcium, mg/dL	8.5 (7.5-9.5)	8.6 (7.8-10)	0.12
Phosphorus, mg/dL	4.8 (3.5 -6.9)	5 (4.4-7)	0.08
Parathormone,pg/dL	460 (97-1700)	230 (15-776)	<0.001
Ferritin, ng/mL	272 (127-1080)	191 (28-1167)	0.008
Albumin, g/dL	3.9 (2.5-4.5)	4 (3.4-4.5)	0.87

Morbidity and mortality

Rates of hospitalization (40% versus 32%, p-value=0.55) and infections (24% versus 12%, p-value) did not differ between the two groups. During the study, there was only one cardiovascular event in the control group and none in the uninsured group of patients. None of the patients died during the study.

## Discussion

In our study, uninsured patients were younger and had fewer comorbidities compared to insured individuals. In many cases, recently arrived immigrants without insurance had a short period of stay in Greece before moving to other countries in the European Union. We have shown that, three months after enrollment, there were no differences in morbidity between uninsured and insured patients. Hospitalization rates were similar in both groups, and no deaths occurred in the uninsured population during the study period.

According to the country, the cost of dialysis for the uninsured population is covered by the health system. Previous studies have shown the need for scheduled dialysis sessions for these patients since it was better for them and resulted in lower cost [8,9]. In Courtsides et al.'s [1] study, undocumented immigrants were less likely to have any pre-dialysis renal care. In addition, the overall costs during initiation of dialysis sessions were higher compared to American dialysis patients [1].

Another critical issue for patients undergoing dialysis is the creation of a mature AVF and the initiation of renal replacement therapy. In the current study, the percentage of uninsured individuals treated by tunneled dialysis catheters was almost double compared to the control group. This observation is in agreement with the study by Raghavan [11] in which the majority of undocumented patients performed hemodialysis through a CVC, while in Chernin et al.'s [2] study, all uninsured patients were treated by tunneled CVCs.

Dialysis-related complications in patients without insurance are documented in several studies [5,8,9,11,12]. In Cervantes et al.'s [8] study in undocumented patients, emergency-only hemodialysis was associated with increased mortality. Additionally, a co-primary endpoint, quality of life, was lower in those patients receiving only emergent dialysis [13,14]. Our findings provide additional support for these observations by showing that, when uninsured patients receive scheduled outpatient dialysis, morbidity remains low and survival outcomes are preserved.

As extensively stated in the study by Moutzouris et al. [15], during 2007 in Greece, uninsured patients with ESRD underwent only emergent hemodialysis sessions. Of note, due to the fact that the cost of dialysis was covered by the national health system, these patients were admitted only to hospitals of the national health system as emergency cases. As a result, patients who need dialysis without insurance were treated in a different hospital every time. This policy resulted in disastrous results, including insufficient dialysis dosing, absence of standard monitoring, and follow-up of drug administration.

During the pandemic and until now, the state has adopted a three-week scheduled dialysis for uninsured individuals in outpatient dialysis units. In our study, we demonstrated that uninsured patients received high-quality treatment, including evaluation and creation of permanent arteriovenous access. This improvement was associated with better laboratory values and enhanced nutritional status, factors known to contribute to improved long-term outcome and survival.

Limitations

The present study has several limitations that should be acknowledged. First, the group of patients is relatively small. As a result, there are restrictions on performing multiple subgroup analyses regarding differences within the two groups. Second, it is a non-randomized, single dialysis unit study. Consequently, the findings are subject to selection bias and may reflect practices and patient characteristics unique to that facility. Finally, the follow-up period, which does not extend beyond two years, limits the ability to evaluate long-term outcomes, such as vascular access durability, cumulative hospitalization burden, or transplant eligibility. Despite these limitations, to our knowledge, it is the first study conducted in the uninsured patients’ population with ESRD undergoing dialysis in our country.

## Conclusions

In this case-control study, we describe our experience treating uninsured patients with ESRD in a private outpatient dialysis unit in our country. Uninsured individuals who received scheduled thrice-weekly hemodialysis demonstrated stable clinical outcomes, including improvement in laboratory parameters and nutritional status, with no observed mortality during the follow-up period. These findings suggest that regularly scheduled dialysis may be associated with favorable short-term outcomes in this population.

These observations should be interpreted in the context of the study’s limited sample size and single-center design. Nevertheless, they highlight the importance of considering the specific needs of uninsured and immigrant patients when planning dialysis care. Policies that facilitate consistent access to scheduled hemodialysis may help support clinical stability and continuity of care in this vulnerable group. Further multicenter studies with larger cohorts and longer follow-up are needed to confirm these associations and to better inform health system strategies aimed at improving the quality of care and long-term outcomes for uninsured patients with ESRD.
